# Predictive Value of the White Blood Cells Count for the Prognosis of Hospitalized Patients with Acute Aortic Dissection

**DOI:** 10.31083/RCM26347

**Published:** 2025-04-18

**Authors:** Yuzhen Suolang, Yizhu Gao, Changli Sun, Shu Zhang

**Affiliations:** ^1^Department of Emergency Medicine, West China Hospital, Sichuan University, 610041 Chengdu, Sichuan, China; ^2^Disaster Medical Center, Sichuan University, 610041 Chengdu, Sichuan, China; ^3^Chengdu Shang Jin Nan Fu Hospital/Shang Jin Hospital of West China Hospital, Sichuan University, 610041 Chengdu, Sichuan, China

**Keywords:** predictive value, white blood cells count, acute aortic dissection, in-hospital mortality

## Abstract

**Objective:**

Our study evaluated the prognostic significance of white blood cells (WBC) count and WBC subsets in relation to the risk of mortality in acute aortic dissection (AAD) patients during their hospital stay.

**Methods:**

We included 833 patients with AAD in this retrospective study. The primary outcome was in-hospital mortality. Cox regression analysis was employed to determine the independent risk factors for mortality in patients with AAD. Amidst the low- and high-WBC groups, we use Kaplan‒Meier survival analysis to compare the cumulative survival rates of patients with AAD.

**Results:**

Within 342 patients with type A AAD, patients belonging to the high-WBC group exhibited a notably higher mortality rate compared to patients in the low-WBC group. Kaplan-Meier analysis exhibited that the patients in high-WBC patients had a significantly higher mortality rate. Multivariable Cox regression analysis demonstrated that an elevated WBC was an independent impact factor of in-hospital mortality of patients with type A AAD (hazard ratio, 2.01; 95% confidence interval (CI): 1.24 to 3.27; *p* = 0.005). Corresponding outcomes were witnessed in 491 patients with type B AAD.

**Conclusions:**

An elevated WBC count was strongly correlated with an elevated risk of mortality in hospitalized patients afflicted with either type A or type B AAD.

## 1. Introduction

Acute aortic dissection (AAD) stems from aortic intima damage due to endogenous or extrinsic factors, permitting blood to course between aortic wall layers [1]. This phenomenon induces the separation of the layers in a proximal or distal direction along the vertical axis [1, 2]. AAD represents a critical ailment commonly encountered within emergency departments, posing a significant risk of sudden death due to vascular rupture or impairment of vital organ function [1]. The incidence of AAD has exhibited a distinctly ascending tendency over the past few years, primarily attributable to the burgeoning elderly population and potential etiological factors such as hypertension and atherosclerosis [3].

Patients with AAD commonly have a notably increased mortality rate [3]. Previous studies indicated that the presence of clinical symptoms in AAD patients leads to an average hourly increase in the mortality rate of approximately 1% to 2% [1, 4]. AAD can be classified into two main categories: Stanford type A, which affects the ascending aorta, and Stanford type B, which does not involve the ascending aorta [1]. For type A AAD, the absence of a prompt diagnosis may result in a mortality rate of 75% in the second week, which could potentially escalate to 90% within a three-month time frame [5]. Consequently, the timely identification and comprehensive assessment of such cases play a pivotal role in determining personalized treatment strategies for individuals with AAD [1, 5]. Some studies have substantiated a robust association between the quantification of white blood cells (WBC) and acute coronary syndrome as well as the overall fatality rate among individuals with peripheral arterial diseases [6, 7, 8, 9]. The incidence and progression of AAD are profoundly influenced by inflammatory reactions [8]. The infiltration of the AAD-affected regions by a substantial quantity of inflammatory cells substantially increases the probability of rate to mortality in patients with AAD [2]. The prognosis of patients with type A AAD may be related to biomarkers that indicate an inflammatory response, such as C-reactive protein (CRP), matrix metalloproteinase 9 (MMP-9), and interleukin 6 (IL-6) [10, 11, 12, 13]. The heightened levels of these inflammatory indicators are significantly correlated with an unfavorable prognosis in patients with type A AAD [12, 14].

WBC, as a simple systemic inflammatory biomarker, has been associated with poor prognosis for patients with cardiovascular diseases [15, 16]. Previous studies showed an elevated WBC was associated with adverse surgical outcomes of type A or type B AAD; however, small sample sizes limited the reliability of the conclusions and there was conflicting evidence, in addition to unclear associations between WBC subsets and mortality in patients with AAD [17, 18, 19, 20]. No comprehensive investigation involving a substantial number of participants has yet been conducted to assess the prognostic significance of WBC count and its subsets in predicting mortality risk among hospitalized patients with AAD. Therefore, this study was conducted to assess the prognostic values of WBC count and WBC subsets for mortality among AAD patients during their hospital stay.

## 2. Materials and Methods

### 2.1 Patient Population

This study was a retrospective cohort study investigating the association of WBC count with in-hospital mortality among patients with AAD. The study was conducted in compliance with the Helsinki Declaration. The Human Ethics Committee of West China Hospital of Sichuan University approved the study protocol (NO.2019-565).

Patients with AAD who were hospitalized in West China Hospital of Sichuan University between January 1st, 2012, and December 31st, 2015, were enrolled in this study. AAD was diagonsed by typical clinical symptoms combined with computed tomography (CT) scanning performed at the time of admission according to the American College of Cardiology (ACC)/American Heart Association (AHA) Guidelines [1]. In this study, 833 AAD patients who are hospitalized to our hospital within 2 weeks after the onset of AAD symptoms were included [1]. Among all patients, 342 patients were diagnosed with type A AAD, while the remaining 491 were diagnosed with type B. Patients were classified into two groups based on their WBC counts, namely, the high-WBC group (≥10 × 10^9^/L) and the low-WBC group (<10 × 10^9^/L).

Patient inclusion criteria:

(1) Patients who were aged 18 years or older;

(2) Patients with a time from symptom onset to hospitalization of ≤2 weeks;

(3) Patients diagnosed with AAD by chest CT angiography in our hospital.

Patient exclusion criteria:

(1) Pregnant women;

(2) Patients diagnosed with AAD resulting from trauma;

(3) Patients who underwent surgical or interventional procedures within one month of AAD onset;

(4) Patients requiring additional operations during their hospitalization period;

(5) Patients who were administered to have some markers such as WBC count, platelet (PLT) count, renal function, and albumin levels;

(6) Patients presenting with comorbidities such as inflammatory, hematological, urological, endocrine, immune system disorders and malignant tumors, which could impact indicators such as the WBC count, the PLT count, renal function, and albumin levels.

### 2.2 Data 

In this present study, we conducted a retrospective analysis of the hospital medical records of all patients diagnosed with AAD and collected comprehensive clinical data from each patient using clinical notes and charts. We systematically compiled demographic data, vital signs, medical history, blood laboratory tests, CT scan results, therapeutic interventions, and in-hospital death. Patient demographic information included age and sex. Vital signs included blood pressure and heart rate. We also documented any medical history of hypertension, diabetes, or atherosclerosis (specifically carotid artery disease) in patients with AAD. Biomarkers related to blood lipids, liver function, and renal function were extracted from the inpatient medical records, as were the WBC and PLT counts upon admission. Therapeutic interventions, encompassing medication, interventional procedures, hybrid operations, and surgical treatments, were also documented. Furthermore, the left ventricular ejection fraction (LVEF) was obtained from inpatient medical records. Lastly, in-hospital mortality data were collected from the clinical medical records.

### 2.3 Endpoints

The primary outcome in the present study was all-cause mortality. We identified deaths by reviewing the clinical records in the hospital.

### 2.4 Statistical Analysis

Statistical analysis was conducted using the software tools SPSS for Windows 21.0 (SPSS, Inc, Chicago, IL, USA) and GraphPad 6.0 (GraphPad Software, Inc, San Diego, CA, USA). The mean ± standard deviation (SD) is used to express normally distributed measurement data, while medians (quartiles) are used for abnormally distributed measurement data. Variance analysis was conducted for data that followed a normal distribution, while the rank sum test was used for data that did not follow a normal distribution to compare the groups. Frequencies or percentages are used to express enumeration data, and comparisons among groups were performed using the χ^2^ test. During the hospitalization period, we performed Cox regression analysis to identify the independent risk factors for mortality in patients with type A and type B AAD. Additionally, we used Kaplan‒Meier survival analysis to compare the cumulative survival rates of patients with type A and type B AAD in the high-WBC and low-WBC groups. The receiver operating characteristic (ROC) curve was utilized to examinvalue tic significance of the WBC count in relation to the mortality of hospitalized patients with AAD. A *p*-value less than 0.05 was deemed to be statistically significant.

## 3. Results

### 3.1 General Data of Patients with AAD Categorized by Various WBC Groups

Among patients with type A AAD, individuals in the high-WBC group demonstrated a higher incidence of hypertension and elevated systolic blood pressure (SBP) and total cholesterol (TC), uric acid, and aspartic transaminase (AST) levels in comparison to those in the low-WBC group. Nevertheless, no statistically significant differences for other variables were found between the two groups, as indicated in Table 1.

**Table 1.** **General information of the patients with AAD in different WBC groups**.

Variables		Type A acute aortic dissection			Type B acute aortic dissection		
		Low-WBC group (n = 125)	High-WBC group (n = 217)	*p* value	Low-WBC group (n = 257)	High-WBC group (n = 234)	*p* value
Age (years)		48 ± 14	49 ± 12	0.580	55 ± 13	50 ± 12	0.000
Male, n (%)		111 (88.8)	198 (91.2)	0.461	217 (84.4)	208 (88.9)	0.149
Smoking, n (%)		56 (44.8)	117 (53.9)	0.104	119 (46.3)	131 (56.0)	0.032
Hypertension, n (%)		60 (48.0)	133 (61.3)	0.017	200 (77.8)	184 (78.6)	0.828
Diabetes, n (%)		4 (3.2)	7 (3.2)	0.990	29 (11.3)	29 (12.4)	0.704
Aortic AS, n (%)		13 (10.4)	26 (12.0)	0.658	58 (22.9)	29 (12.6)	0.003
Heart rate (beats/min)		86 ± 15	86 ± 16	0.996	81 ± 14	87 ± 16	0.000
SBP (mmHg)		130 ± 24	139 ± 28	0.003	145 ± 25	154 ± 29	0.000
DBP (mmHg)		74 ± 17	77 ± 19	0.068	87 ± 16	92 ± 21	0.004
TGs (mmol/L)		3.56 ± 0.89	3.66 ± 0.97	0.356	2.56 ± 1.48	4.90 ± 2.67	0.278
TC (mmol/L)		1.19 ± 0.70	1.52 ± 1.10	0.002	2.74 ± 1.74	2.58 ± 1.56	0.309
LDL-C (mmol/L)		2.02 ± 0.76	2.06 ± 0.79	0.684	3.11 ± 13.54	2.23 ± 0.70	0.348
HDL-C (mmol/L)		0.99 ± 0.45	0.95 ± 0.42	0.434	1.00 ± 0.40	1.09 ± 0.65	0.063
Creatinine (µmol/L)		123 ± 197	131 ± 120	0.664	101 ± 108	110 ± 114	0.399
Urea nitrogen (mmol/L)		7.72 ± 5.93	9.15 ± 7.47	0.075	6.70 ± 5.50	7.17 ± 5.38	0.340
Uric acid (mmol/L)		311 ± 124	363 ± 175	0.002	302 ± 107	320 ± 124	0.084
Albumin (g/L)		34 ± 6	34 ± 6	0.816	34 ± 6	36 ± 6	0.019
AST		47 ± 112	266 ± 1054	0.004	43 ± 189	62 ± 221	0.306
PLT (10^9^/L)		184 ± 91	168 ± 86	0.105	204 ± 92	190 ± 88	0.109
LVEF (%)		62.0 ± 8.4	61.6 ± 7.8	0.658	62.4 ± 7.5	62.0 ± 8.4	0.629
Treatment methods		-	-	0.921	-	-	0.890
	Medication, n (%)	64 (51.2)	116 (53.5)		70 (27.2)	65 (27.8)	
	Hybrid operation/ Intervention^*^, n (%)	8 (6.4)	13 (6.0)		178 (69.3)	159 (67.9)	
	Surgery, n (%)	53 (42.4)	88 (40.6)		9 (3.5)	10 (4.3)	

AAD, acute aortic dissection; WBC, white blood cells; AS, atherosclerosis; SBP, systolic blood pressure; DBP, diastolic blood pressure; TGs, triglycerides; TC, total cholesterol; LDL-C, low density lipoprotein cholesterol; HDL-C, high density lipoprotein cholesterol; AST, aspartic transaminase; PLT, platelets; LVEF, left ventricular ejection fraction. ^*^Hybrid operations are exclusively conducted for Type A AAD, whereas interventions are specifically for Type B AAD.

Among patients with type B AAD, those in the high-WBC group were younger, had a higher incidence of smoking and aortic atherosclerosis (AS), and had higher average heart rate (HR), SBP, diastolic blood pressure (DBP), and albumin level compared to those in the low-WBC group. Nevertheless, no statistically significant differences in the remaining variables were observed between the two groups, as indicated in Table 1.

### 3.2 The Variation in the Mortality Rate in Hospitalized Patients with AAD Across Different WBC Groups

**Fig. 1.**
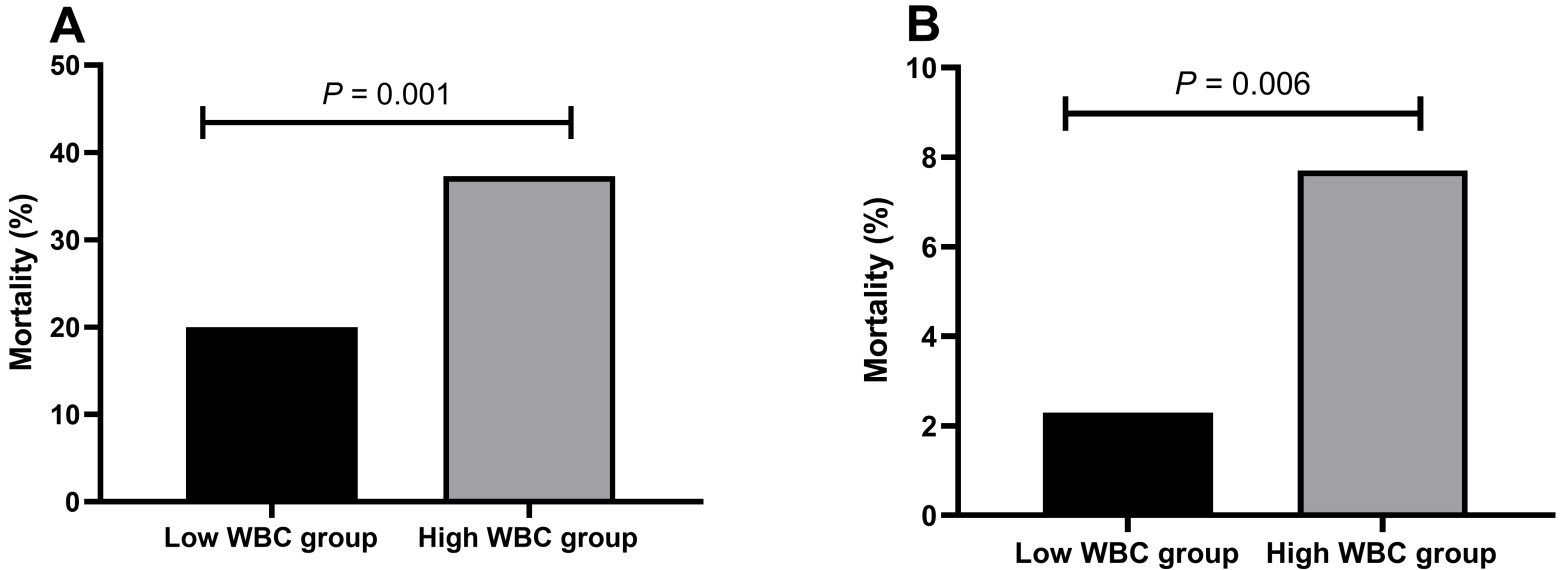
**The mortality rates of patients with AAD in different WBC groups**. (A) The mortality rate of patients with type A aortic dissection in the high-WBC and low-WBC groups. (B) The mortality rate of patients with type B aortic dissection in the high-WBC and low-WBC groups. AAD, acute aortic dissection; WBC, white blood cells.

Among patients with type A AAD, individuals in the high-WBC group demonstrated a notably increased mortality rate (37.3% vs. 20.0%) in comparison to patients in the low-WBC group, with a statistically significant difference (*p *= 0.001). Similarly, among patients diagnosed with type B AAD, those in the high-WBC group exhibited a significantly higher mortality rate (7.7% vs. 2.3%) than patients in the low-WBC group (*p* = 0.006, Fig. 1).

### 3.3 Kaplan‒Meier Survival Analysis of Patients with AAD in Different WBC Groups

**Fig. 2.**
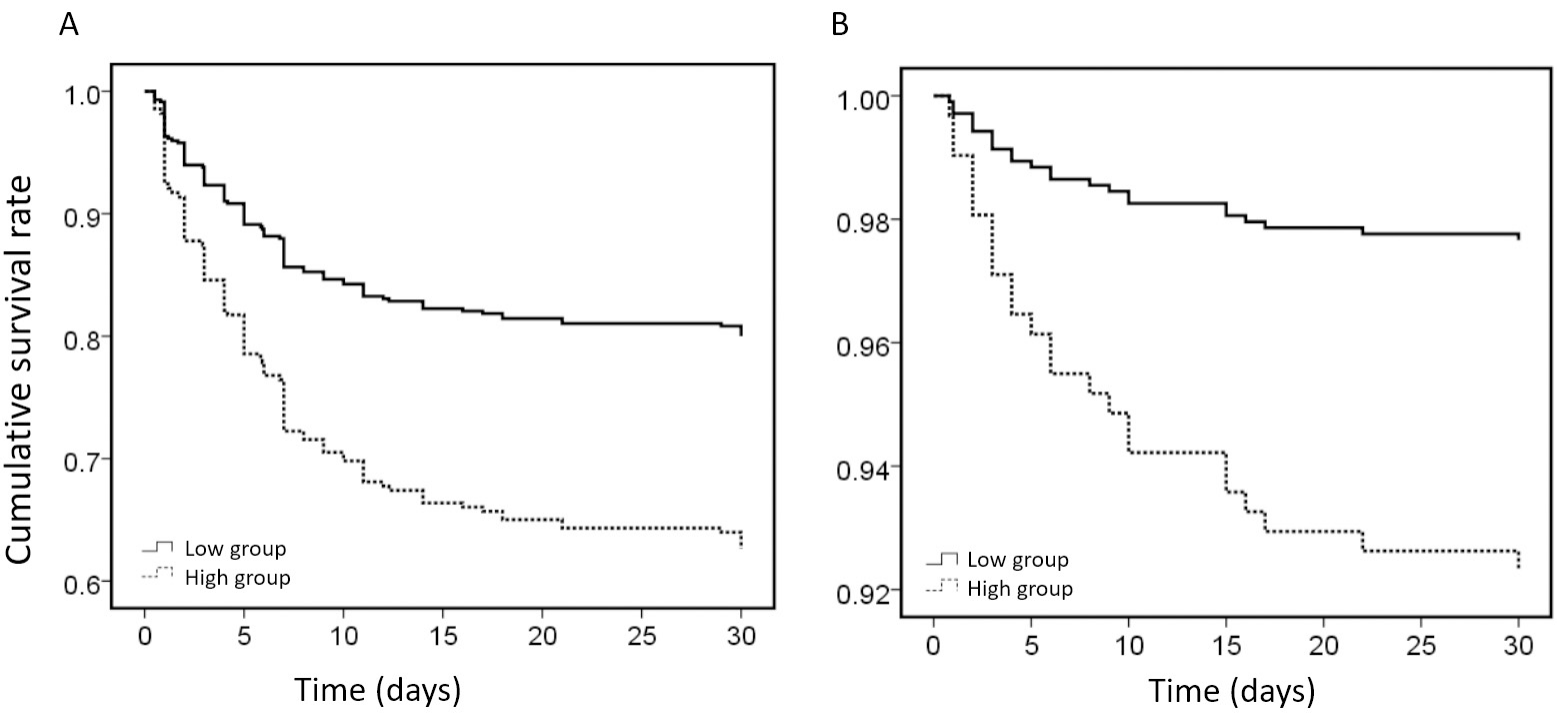
**Kaplan‒Meier survival analysis of patients with AAD in different WBC groups**. (A) Kaplan‒Meier survival analysis of patients with type A AAD in different WBC groups. (B) Kaplan‒Meier survival analysis of patients with type B AAD in different WBC groups. AAD, acute aortic dissection; WBC, white blood cells.

Patients with type A AAD in the high-WBC group exhibited a significantly lower survival rate when compared to those in the low-WBC group (*p *= 0.001). Similarly, patients with type B AAD in the high-WBC group demonstrated a significantly lower survival rate than those in the low-WBC group (*p* = 0.006, Fig. 2).

### 3.4 Cox Regression Analysis of the Mortality of Hospitalized Patients with Type A AAD

The univariate Cox regression analysis revealed a significant correlation between the mortality risk of hospitalized patients with type A AAD and several factors, including a WBC count ≥10 × 10^9^/L, age, the PLT count, uric acid levels, hemoglobin (Hb) levels, and treatment methods. Furthermore, the multivariate Cox regression analysis identified a WBC count ≥10 × 10^9^/L (hazard ratio [HR]: 2.01, 95% confidence interval [CI]: 1.24–3.27, *p* = 0.005), the PLT count (HR: 0.995, 95% CI: 0.992–0.999, *p* = 0.004), and treatment methods (hybrid operation vs. medication: HR: 0.08, 95% CI: 0.01–0.61, *p* = 0.005; surgery vs. medication: HR: 0.15, 95% CI: 0.08–0.27, *p* = 0.000) as independent factors associated with mortality in hospitalized patients with type A AAD (Table 2).

**Table 2.** **Cox regression analysis of factors related to the mortality of hospitalized patients with type A AAD**.

Variables	Univariate regression analysis			Multivariate regression analysis		
	HR	95% CI	*p*	HR	95% CI	*p*
WBC count ≥10 × 10^9^/L	2.08	1.32–3.25	0.001	2.01	1.24–3.27	0.005
Age	1.02	1.005–1.036	0.008	1.01	0.99–1.03	0.261
PLT count	0.994	0.991–0.997	0.000	0.995	0.992–0.999	0.004
Uric acid level	1.001	1.000–1.002	0.030	0.999	0.998–1.000	0.239
Hb level	1.01	1.00–1.02	0.050	1.004	0.993–1.014	0.504
Treatment methods	-	-	0.000	-	-	0.000
Medication	1	-	-	1	-	-
Hybrid operation	0.07	0.01–0.49	0.008	0.08	0.01–0.61	0.005
Surgery	0.15	0.08–0.25	0.000	0.15	0.08–0.27	<0.001

AAD, acute aortic dissection; WBC, white blood cells; PLT, platelet; Hb, hemoglobin; HR, hazard ratio; CI, confidence interval.

### 3.5 Cox Regression Analysis of the Mortality of Hospitalized Patients with Type B AAD

Based on the findings of the univariate Cox regression analysis, a notable association was observed between the mortality risk of hospitalized patients diagnosed with type B AAD and various factors, including an elevated WBC count (WBC count ≥10 × 10^9^/L), the presence of aortic AS, heart rate, low density lipoprotein cholesterol (LDL-C), creatinine, urea nitrogen, uric acid, albumin, and AST levels, and the employed treatment modalities. A multivariate Cox regression analysis identified aortic AS (HR 3.89, 95% CI: 1.21–12.57, *p* = 0.023) and a WBC count ≥10 × 10^9^/L (HR 4.52, 95% CI: 1.25–16.34, *p* = 0.021) as the primary factors contributing to the mortality of hospitalized patients diagnosed with type B AAD (Table 3).

**Table 3.** **Cox regression analysis of factors related to the mortality of hospitalized patients with type B AAD**.

Variables	Univariate regression analysis			Multivariate regression analysis		
	HR	95% CI	*p* value	HR	95% CI	*p* value
WBC count ≥10 × 10^9^/L	3.39	1.34–8.53	0.006	4.52	1.25–16.34	0.021
Aortic AS	2.33	1.01–5.56	0.049	3.89	1.21–12.57	0.023
Heart rate	1.05	1.02–1.07	0.000	1.02	0.99–1.05	0.237
LDL-C	0.35	0.17–0.73	0.005	0.59	0.26–1.40	0.235
Creatinine	1.003	1.002–1.004	0.000	1.000	0.996–1.004	0.941
Urea nitrogen	1.08	1.06–1.11	0.000	1.04	0.98–1.11	0.174
Uric acid	1.006	1.003–1.009	0.000	1.003	0.999–1.008	0.190
Albumin	0.94	0.89–0.99	0.029	0.96	0.88–1.04	0.303
AST	1.001	1.000–1.002	0.001	1.000	1.000–1.001	0.336
Treatment methods	-	-	0.000	-	-	0.250
Medication	1	-	-	1	-	-
Intervention	0.13	0.05–0.34	0.000	0.39	0.13–1.22	0.107
Surgery	0.39	0.05–2.96	0.364	0.46	0.04–5.24	0.532

WBC, white blood cells; LDL-C, low-density lipoprotein cholesterol; AST, aspartic transaminase; AS, atherosclerosis; AAD, acute aortic dissection; HR, hazard ratio; CI, confidence interval.

### 3.6 The ROC Curve of the WBC Count and its Subsets for the Prediction of Death in Hospitalized Patients with AAD

ROC analysis revealed that the area under the curve (AUC) values of the WBC, neutrophil (NEUT), lymphocyte (LYM), and monocyte (MONO) counts for predicting the outcomes of hospitalized patients with type A AAD were 0.615 (95% CI: 0.552–0.678; *p* = 0.001), 0.620 (95% CI: 0.557–0.682; *p* = 0.000), 0.500 (95% CI: 0.433–0.568; *p* = 0.989), and 0.597 (95% CI: 0.534–0.659; *p* = 0.004), respectively. Based on the analysis of the ROC curve, it was determined that the AUC values for hospitalized patients diagnosed with type A AAD, as predicted by the WBC, NEUT, LYM, and MONO counts, were 0.762 (95% CI: 0.657–0.868; *p* = 0.000), 0.762 (95% CI: 0.658–0.866; *p* = 0.000), 0.514 (95% CI: 0.373–0.655; *p* = 0.816), and 0.644 (95% CI: 0.525–0.763; *p* = 0.644), respectively (Table 4).

**Table 4.** **The predictive value of the WBC count and its subsets for the mortality of hospitalized patients with AAD**.

	Type A acute aortic dissection			Type B acute aortic dissection		
	AUC	95% CI	*p*	AUC	95% CI	*p*
WBC count	0.615	0.552–0.678	0.001	0.762	0.657–0.868	0.000
Neutrophil count	0.620	0.557–0.682	0.000	0.762	0.658–0.866	0.000
Lymphocyte count	0.500	0.433–0.568	0.989	0.514	0.373–0.655	0.816
Monocyte count	0.597	0.534–0.659	0.004	0.644	0.525–0.763	0.017

AAD, acute aortic dissection; AUC, area under the curve; WBC, white blood cells; CI, confidence interval.

## 4. Discussion

### 4.1 Clinical Risk Stratification and Predictive Factors of Aortic Dissection

AAD is a cardiac condition with high severity, marked by a notable mortality rate. The rapid economic growth in China has led to a gradual comorbid diseases such as hypertension, diabetes, hypercholesteremia, and atherosclerosis. Consequently, the incidence of AAD has also exhibited an upward trend over the years [1, 21, 22]. Enhancing prognosis necessitates timely identification and diagnosis and tailored interventions, given the substantial fatality rate associated with AAD [1]. Evaluation of the risk level in individuals with AAD and the implementation of tailored treatment interventions according to their risk levels are imperative in clinical practice to mitigate the mortality rate and long-term adverse consequences linked to AAD [1]. Previous studies have demonstrated that inflammation not only serves as the primary pathological alteration in atherosclerosis but also exerts a substantial influence on the initiation and progression of AAD [8, 23]. Biomarkers indicating an inflammatory response, such as hypersensitive C-reactive protein (hs-CRP) levels [12, 24], the WBC count [20, 25], D-dimer levels [12, 26], and the PLT count forecast the prognosis of AAD patients [27].

Li *et al*. [28, 29] conducted a study wherein patients diagnosed with AAD were included as participants. The study findings indicated that the thrombosis-inflammation response plays a substantial role in the initiation and advancement of AAD. To assess this response, a scoring system was developed by calculating the ratio of the WBC count and mean platelet volume (MPV) to the PLT count upon patient admission. The results demonstrated that this scoring system holds predictive value for both complications during hospitalization and long-term mortality. Based on the study conducted by Wen *et al*. [30], it was observed that individuals with AAD who died during hospitalization, as well as those with pleural effusion, demonstrated heightened plasma CRP levels and WBC counts. Additionally, a negative association was identified between the plasma CRP level and WBC count and the duration from symptom onset to hospital admission. During the period of hospitalization, a significant correlation was observed between mortality and several factors, including age (65 years or older), an elevated CRP level of 12.05 mg/L or higher, an increased WBC count of 12.16 × 10^9^/L or higher, an aortic diameter measuring 48 mm or larger, the presence of pleural effusion, and a DBP exceeding 105 mmHg. This finding was determined by utilizing multivariate regression analysis.

Sbarouni *et al*. [31] observed that patients with AAD demonstrated significantly elevated WBC counts, NEUT to LYM ratios, and D-dimer levels compared to individuals with chronic arterial aneurysms and healthy controls. Among the patients diagnosed with AAD, those who died exhibited a noteworthy increase in the WBC count and D-dimer concentration. Furthermore, the NEUT to LYM ratio proved to be a valuable diagnostic and therapeutic tool for promptly identifying and managing AAD patients. The combination of elevated levels of NEUTs and decreased levels of LYMs (increased neutrophil to lymphocyte ratio (NLR)) has been demonstrated to promote atherosclerosis, arterial stiffness and aortic disease in multiple studies [32, 33, 34]. However, the prognostic implications of specific subsets of WBC, such as NEUTs, MONOs, and LYMs, were not addressed in this study regarding the prognosis of patients with AAD. Additionally, the study did not investigate the pathophysiological mechanisms underlying the involvement of these subsets of WBC in patients with AAD.

### 4.2 Predictive Value of the WBC Count for the Mortality of Patients with Aortic Dissection

This study examined the influence of the WBC count and its subsets on the prognosis of hospitalized patients with AAD. The participants in this research were individuals diagnosed with AAD, and the results demonstrated a noteworthy difference between patients in the high-WBC group and those in the low-WBC group for both type A and type B AAD. Based on the Kaplan‒Meier analysis indicated a notable difference in survival rate between patients in the high-WBC group and those in the low-WBC group within both the type A and B cohorts. These findings align with prior investigations [4, 18, 19, 20, 28, 29], substantiating the notion that an elevated WBC count serves as an indicator of unfavorable clinical outcomes in individuals diagnosed with AAD. Our study utilized multivariate regression analysis to investigate the association between the WBC count and its subsets (the NEUT, LYM, and MONO counts) in the context of inflammatory reactions. The results indicated that patients diagnosed with AAD who exhibited a higher level of WBC had a significantly increased risk of mortality during their hospitalization period. In patients diagnosed with type B AAD, a higher WBC count of 10 × 10^9^/L was identified as a significant predictor of mortality during hospitalization.

In addition, WBC count has been recognized determinant independent prognostic factor for various cardiovascular conditions, including acute ST-elevation myocardial infarction (STEMI), ischemic cardiomyopathy (ICM), and aneurysm [15, 35]. Acute coronary syndrome and acute aortic syndrome (AAS) are characterized by a sustained inflammatory process. The rupture of plaque in adjacent blood vessels, influenced by various factors, leads to thrombosis or detachment of the innermost layer of the vessels, ultimately resulting in aortic dissection [36]. The occurrence of acute complications in blood vessels, induced by chronic vascular inflammation, triggers the activation of potential inflammatory responses and the development of aseptic inflammation. The development and prognosis of patients with AAD can be influenced by multiple pathways, including the interaction of WBC with various factors, such as PLTs, tissue factors, and fibrins. In addition to producing numerous, inflammatory factors, WBC play a significant role in the progression and outcomes of vascular lesions in individuals with AAD [37, 38, 39].

## 5. Conclusions

WBC count has particular significance in predicting the outcomes of hospitalized patients with AAD. A high WBC was associated with an increased risk of mortality in hospitalized patients with either type A or type B AAD. This association indicated a high WBC was a risk factor for mortality during hospitalization. Furthermore, the subsets of WBC, including NEUTs and MONOs, also possess predictive significance for the mortality of hospitalized patients with AAD. Elevated NEUT and MONO counts may contribute to the predictive value of the WBC count in AAD.

## Availability of Data and Materials

The datasets used and analyzed during the current study are available from the corresponding author on reasonable request.
